# Efficiency Improvement for Chipless RFID Tag Design Using Frequency Placement and Taguchi-Based Initialized PSO

**DOI:** 10.3390/s24144435

**Published:** 2024-07-09

**Authors:** Cong-Cuong Le, Trung-Kien Dao, Ngoc-Yen Pham, Thanh-Huong Nguyen

**Affiliations:** International Research Institute MICA, School of Electrical and Electronic Engineering, Hanoi University of Science and Technology, Hanoi 100000, Vietnam; yen.phamthingoc@hust.edu.vn

**Keywords:** chipless RFID, radar cross section (RCS), particle swarm optimization (PSO), Taguchi method (TM)

## Abstract

Frequency encoding chipless Radio Frequency Identification (RFID) tags have been frequently using the radar cross section (RCS) parameter to determine the resonant frequencies corresponding to the encoded information. Recent advancements in chipless RFID design have focused on the generation of multiple frequencies without considering the frequency position and signal amplitude. This article proposes a novel method for chipless RFID tag design, in which the RCS response can be located at an exact position, corresponding to the desired encoding signal spectrum. To achieve this, the empirical Taguchi method (TM), in combination with particle swarm optimization (PSO), is used to automatically search for optimal design parameters for chipless RFID tags with a fast response time, to comply with the frequency encoding requirements in the presence of the mutual coupling effect. The proposed design method is validated using I-slotted chipless tag structures that are fabricated and measured with different sets of resonant frequencies.

## 1. Introduction

Chipless Radio Frequency Identification (RFID) technology has been attracting significant attention [1,2,3]. Unlike traditional RFID, this technology uses passive elements to encode data in the time or frequency domains [4]. Without using an integrated chip (IC) for data storage and signal modulation, the tag price can be reduced. Moreover, the planar tag structure can even be fabricated using printing technology, which is simple and cost-effective [5,6,7]. These features enable chipless RFID tags to replace barcodes and QR codes in the future.

**Figure 1:**
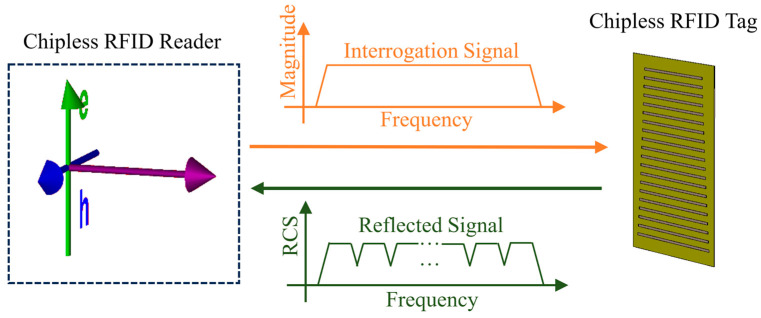
Backscattering chipless RFID system.

However, the main limitation of chipless RFID technology is its lower encoding capacity in terms of bits compared with other technologies [8]. To address this problem, much research has been devoted to design and data encoding methods [3]. Frequency-domain chipless tags have been shown to achieve a higher encoding capacity than time-domain tags [9]. Frequency-based tags can be categorized into two types: retransmitting and backscattering tags. A tag of the first type can retransmit the interrogating signal from the reader after being modulated by a series of passive elements, such as band-stop filters with narrow bandwidths [7,9,10,11]. Owing to the presence of transmitting and receiving antennas, this tag often has a larger size, which is an obvious disadvantage. In the second tag type, the tag scatters back the electromagnetic waves received from the reader [6,8]. This type does not require a pair of transceiver antennas; thus, it has a smaller size, and the coding capacity is dominant over the first type with the same tag dimension. The backscattering signal to the reader of the backscatter tag in a chipless RFID system is shown in Figure 1.

In the system illustrated in Figure 1, after the reader emits a broadband electromagnetic signal to the tag, the backscattering signal received by the reader consists of three main components, represented by the following equation [12,13]:

(1) $$y_{rx}\left(f\right)=y_{r}\left(f\right)+y_{s}\left(f\right)+y_{t}\left(f\right)$$

where $y_{r}\left(f\right)$ is the signal reflected by the receiving antenna owing to impedance mismatch and attenuation over time, $y_{s}\left(f\right)$ is the reflected signal at the same frequency and received by the receiving antenna, and $y_{t}\left(f\right)$ is the signal generated by the resonant elements on the tag and carries the encoding information of the tag. Different tags can create different sets of resonant frequencies to encode the data. To create these different resonant frequencies, the tag must include passive resonant elements that function like narrowband antennas. Consequently, decoding the encoded data from the tag becomes a matter of determining and analyzing the $y_{t}\left(f\right)$ component in the electromagnetic signal scattered back to the reader. The determination of the resonant frequencies of $y_{t}\left(f\right)$ is conducted with help of the Fourier transform and subsequently locates the peak points on the corresponding frequency spectrum. Nevertheless, the challenge of this method is to ascertain the proper starting time of $y_{t}\left(f\right)$ to eliminate the signal component that does not carry the encoded information. Additionally, this signal component possesses an insubstantial amplitude; thus, it is difficult to measure it accurately.

**Figure 2:**
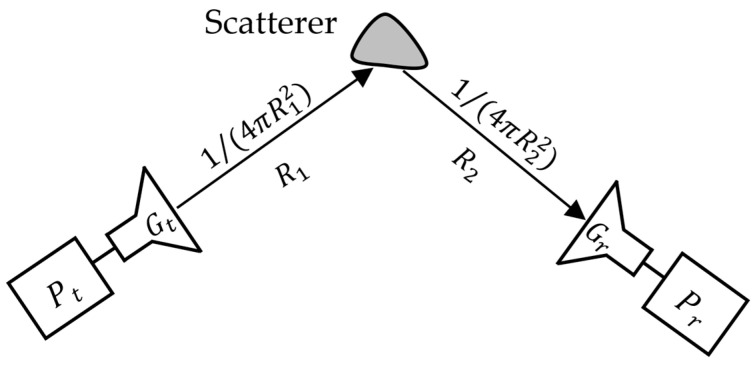
Principal of measuring RCS of the object.

Another widely used method to solve the above-mentioned problems is based on analyzing the radar cross section (RCS) response [14], as illustrated in Figure 2. Accordingly, the RCS of the object is a scalar quantity and represents the relationship between the signal power $P_{r}$ received by the system after sending a signal with transmitting power $P_{t}$ as follows:

(2) $$\sigma =\frac{P_{r}}{P_{t}}\frac{{\left(4\pi \right)}^{3}R_{1}^{2}R_{2}^{2}}{{G_{t}G}_{r}\lambda^{2}}$$

where $G_{t}$ and $G_{r}$ are the gains of the transmitting and receiving antennas, respectively; $R_{1}$ and $R_{2}$ are the distances between the object and transmitting and receiving antennas, respectively; and λ is the wavelength of the emitting signal.

When applied to a transceiver system using one antenna, we obtain $G_{t}=G_{r}=G$ and $R_{1}=R_{2}=R$. Then, Equation (2) can be rewritten as Equation (3) as follows:

(3) $$\sigma =\frac{P_{r}}{P_{t}}\frac{{\left(4\pi \right)}^{3}R^{4}}{G^{2}\lambda^{2}}$$

The RCS depends on the frequency, and the coefficient $\frac{P_{r}}{P_{t}}$ is the square of the reflection coefficient $S_{11}$ for a single antenna; hence, a minimum of σ corresponds to a generated resonant frequency. However, to analyze the RCS of the chipless RFID tags, it should be noted that the received power $P_{r}$ is composed of the powers of the three signal components in Equation (1). Therefore, to properly identify the resonant frequencies to encode the tag’s data, the RCS must be determined with a power that does not contain the two signals $y_{r}\left(f\right)$ and $y_{s}\left(f\right)$. In addition, the background noise must also be cancelled. In this case, the RCS value is calculated indirectly through the reflection coefficient $S_{11}$ according to the following equation [15]:

(4) $$\sigma^{tag}={\left(\frac{S_{11}^{tag}-S_{11}^{iso}}{S_{11}^{ref}{-S}_{11}^{iso}}\right)}^{2}\sigma^{ref}$$

where $S_{11}^{tag}$, $S_{11}^{iso}$, and $S_{11}^{ref}$ are the reflection coefficients $S_{11}$ when the tag is present, the tag is absent, and the reference tag is made of metal with the same dimension, respectively; $\sigma^{ref}$ is the RCS value of the reference tag of width a, length b, and wavelength λ according to Equation (5).

(5) $$\sigma^{ref}=4\pi \frac{a^{2}b^{2}}{\lambda^{2}}$$

The determination of the resonant frequencies of a chipless RFID tag using the RCS value is simple and accessible because the RCS parameter can be directly collected in common microwave simulation software, such as CST Microwave Studio 2024, Altair Feko 2022, and Agilent HFSS 2023 R2, and the RCS measurement can be derived from the measured reflection coefficient $S_{11}$.

To improve the encoding capacity of chipless RFID tags, several data encryption methods based on resonant frequencies were reported in [3]. Consequently, the basic mechanism for improving the encoding capacity is to increase the number of resonating elements in the tag structure. However, to properly encode in the frequency domain, the following two requirements must be satisfied:

(i) The resonant frequencies corresponding to the elements can vary only within a limited frequency range when the tag adds or removes other resonant elements.
(ii) The difference in terms of RCS amplitude at resonant and non-resonant frequencies must be large enough in order to make the minima distinguishable.

The mutual coupling among resonant elements on the tag may lead to a frequency shift [6,8], which cannot ensure the competence of the two above-mentioned requirements. A painstaking analysis of the influence of mutual coupling on encoding resonant frequencies was presented in [16], which showed that this influence is greater when the frequency difference is smaller.

**Figure 3:**
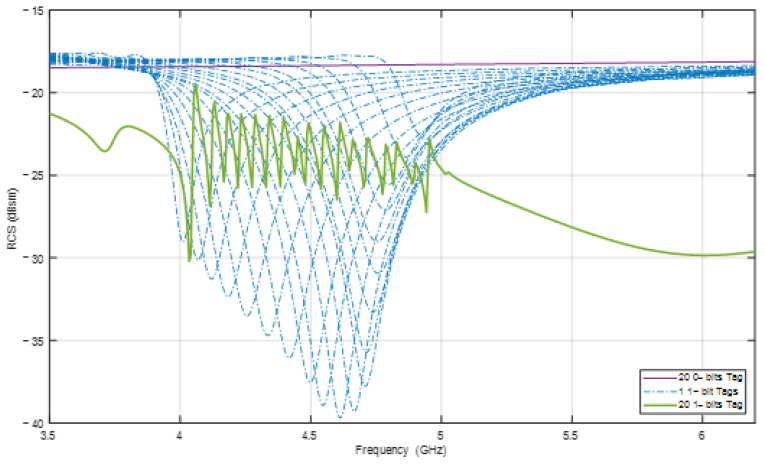
Example RCS response of tags with different numbers of resonant elements.

An example is shown in Figure 3, which simulates the RCS response of a tag with 20 resonant elements conveyed by the green solid line, whereas the dashed lines correspond to 20 tags that only have a single resonator. It is clear that a tag with 20 elements can create 20 resonant frequencies at 20 minima. However, the corresponding frequencies of these minima are shifted compared with the original ones in the dashed lines. Moreover, the deviation is different for each resonator and the shifted frequencies may overlap with the others. This phenomenon is caused by the mutual coupling effect of each element with the other elements. In addition, the amplitudes at the minimum points are also changed to values that are difficult to distinguish. Table 1 lists the exact values of the original frequencies when the elements work individually together with the shifted frequencies and the frequency deviation when all the elements are present on the same tag. Consequently, mutual coupling between the resonant elements on the tag decreases the reliability of the encoded data. This is an important problem that must be investigated and addressed.

Theoretically, accurately computing the mutual coupling between resonant elements on a tag is highly challenging. The first reason is that there is no waveguide port for the resonant elements; thus, modeling each element to calculate the electromagnetic radiation is only approximate. However, the method of calculating the mutual coupling between antennas is also not precise [17,18,19]. Current research only focuses on proposing designs capable of creating more resonant frequencies per unit area, but has not paid attention to thoroughly handling this problem by compensating for frequency shifting.

In this paper, we propose a novel method to design chipless RFID tags, in which the mutual coupling phenomenon is addressed radically, while satisfying the two above-mentioned design requirements (i) and (ii). The principle is to utilize the particle warm optimization (PSO) algorithm [20] and the Taguchi empirical optimization method (TM) [21] to automatically search and optimize the design parameters of a tag.

The remainder of this paper is organized as follows. Section 2 introduces our proposed methodology for tag design using PSO and the Taguchi method. In Section 3, the proposed approach is applied to the design of three tags, each with a different frequency set, to demonstrate its advantage over the traditional approach. This is followed by Section 4, which presents the experimental results with tags fabricated using the designed parameters obtained in Section 3. Finally, Section 5 concludes the paper.

## 2. Proposed Approach

**Figure 4:**
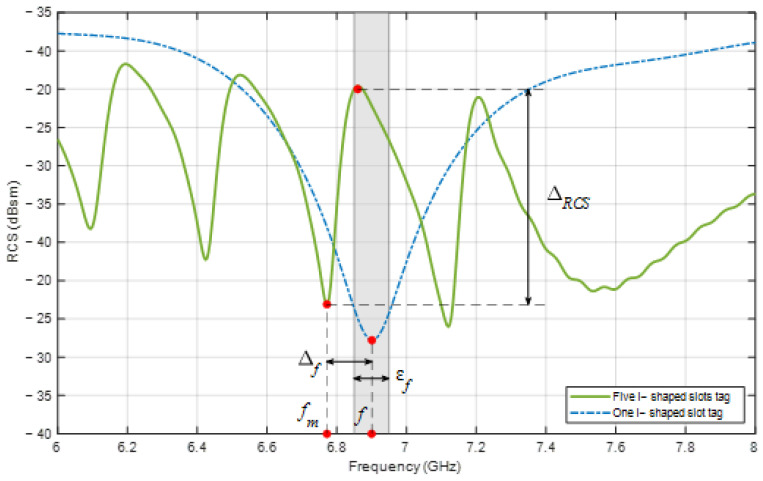
Parameters related to frequency shift caused by the mutual coupling.

The resonant frequency response shift, owing to the mutual coupling effect on the tag, is discussed in Section 1. This shift is the highest when all resonant elements are present in the tag. Figure 4 illustrates the frequency deviation parameters of an encoding tag with five elements, where f is the original frequency when there is no mutual coupling, $f_{m}$ is the frequency achieved by the same element when mutual coupling is present, ${∆}_{f}$ is the frequency deviation, $\varepsilon_{f}$ indicates the accepted frequency deviation limit so that $f_{m}$ is considered to satisfy the encoding requirements, that is, it is distinguishable by the reader, and ${∆}_{RCS}$ is the difference in the RCS value between two consecutive extreme points.

**Figure 5:**
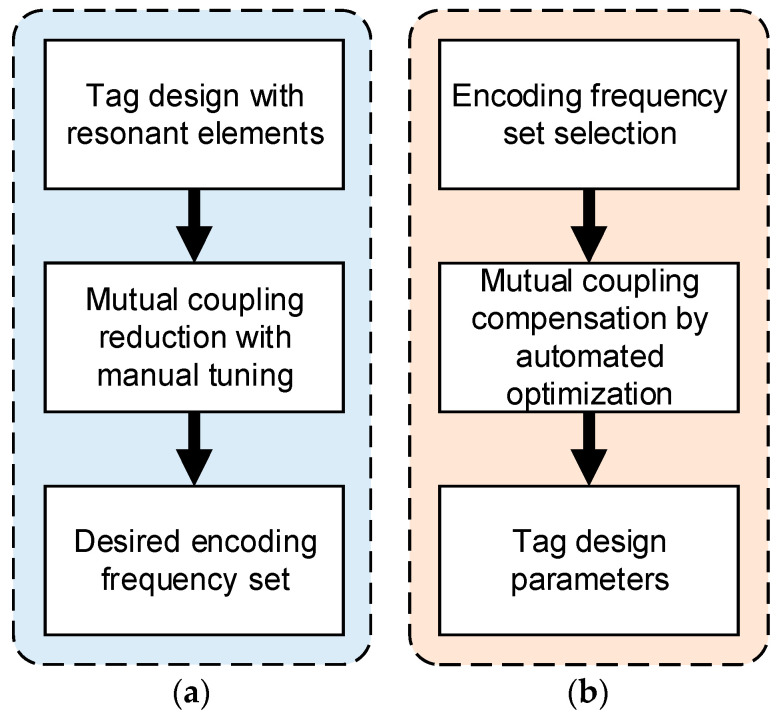
Tag design process in the presence of mutual coupling: (**a**) traditional; (**b**) proposed.

The value of $\varepsilon_{f}$ is determined to be half the spacing between two adjacent resonant frequencies. For example, as shown in Figure 4, $f_{m}$ is not located within the accepted range; hence, it does not satisfy the encoding constraints. Furthermore, ${∆}_{RCS}$ must be higher than a threshold such that the identification of the resonant frequency corresponding to the minimum point is reliable. Consequently, a tag design satisfies the data encoding requirements only if ${∆}_{f}$ and ${∆}_{RCS}$ for all resonant frequencies satisfy the predefined constraints under the impact of mutual coupling. To accomplish this, it is necessary to adjust the design parameters of the resonant elements of the tag for each specific encoding case. As discussed in Section 1, the accurate computation of the design parameters is not trivial. There are two main solutions to reduce the influence of mutual coupling between elements: to survey and rearrange the resonant electromagnetic components [6,22,23,24] or to add a ground plane for isolation [8,25,26]. A typical tag design process using a solution that reduces the influence of mutual coupling is illustrated in Figure 5a.

It can be seen that the investigation of the location of resonant or isolation elements to reduce the influence of mutual coupling is time consuming. Furthermore, reducing or canceling out the mutual coupling is not always feasible, especially when integrating a large number of elements on a tag with a limited size, or when complex tag types are used; this approach is not general and may not yield satisfactory results. Based on this irrelevant consequence, in this article, a novel chipless RFID tag design solution is proposed, as shown in Figure 5b. Accordingly, the first step is to pre-select the desired encoding frequency set and then use the PSO algorithm to automatically find the design parameters corresponding to the influence of mutual coupling. The outcome of the optimization process is a tag design parameter set that satisfies the initially defined frequency encoding error limit. The proposed design method is highly reliable and general because the encoding frequencies can be pre-specified and ensure the encoded data, and is called the Frequency Placement Coding (FPC) method [16].

**Figure 6:**
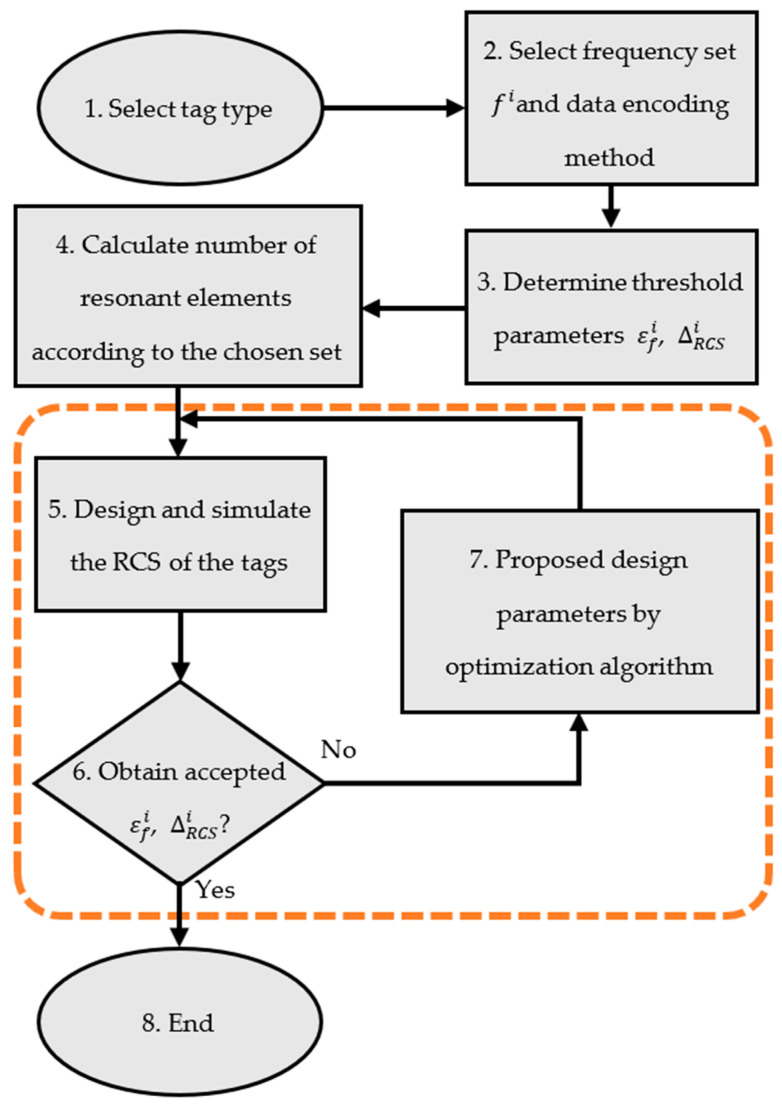
Chipless tag design using optimization algorithm.

Figure 6 shows the detailed process of the tag design steps based on the optimization algorithm according to the proposed method, as follows:

- Step 1: Select the backscattering tag type. Different tag structures with multiple resonant frequencies, small sizes, and low influence of mutual coupling have been introduced [27]. However, with the proposed design method, mutual coupling can easily be compensated; hence, the structure type can be freely specified by the user.
- Step 2: Select the encoding resonant frequency set $F=\left\{f^{i}\right\}$. These frequencies depend primarily on the operating range of the reader antenna and the size limit of the tag. In addition, it is necessary to select a data encoding method that uses these frequencies. Many data encoding methods for chipless RFID tags have been reported, of which the basic method is OOK (On–Off Keying), where each resonant frequency corresponds to a binary bit. The data bit has a logic value of “1” when the corresponding resonant element exists and has a logic value of “0” in the opposite case. In this method, as the number of resonant elements on the tag increases, the physical distance between them decreases and the difference in the encoded resonant frequencies also diminishes. This also increases the influence of mutual coupling between the elements, leading to encoded data corruption. Recently, the Frequency Shift Coding (FSC) method has allowed us to limit the increase in the number of resonant elements. In this method, the parameters of each element are adjusted to resonate at a specific frequency among a predefined set of encoded resonant frequencies. For this reason, the frequency combinations from frequency set F can be realized with a small number of resonant elements on the tag.
- Step 3: Determine the threshold parameters $\varepsilon_{f}^{i}$ and ${∆}_{RCS}^{i}$ to evaluate the quality of the design parameter sets after obtaining the RCS response, as shown in Figure 4. Increasing the number of encoding frequencies in F in the working range makes $\varepsilon_{f}^{i}$ smaller, and it is more difficult to find design parameters. To overcome this problem, a robust optimization method is required, as discussed in Section 2. The value of ${∆}_{RCS}^{i}$ depends on the accuracy of the measurement system, ability of the resonant structure to backscatter electromagnetic signals, tag material, and quality requirements imposed on the RCS signals.
- Step 4: From the set of frequencies, the apply the FSC method to specify the frequency combinations, noting that each frequency corresponds to a resonant element on the tag. Theoretically, each resonant element can be treated as an antenna; therefore, the design parameters of the magnetic part can be calculated according to antenna theory. However, the antenna model of the chipless RFID tag resonator element does not have a fixed waveguide port, and the computation of the design parameters is approximate. This discrepancy requires calibration and is handled optimally in subsequent steps.
- Step 5: Design and simulate the RCS response of the tag using specialized software such as CST Microwave Studio 2024, HFSS 2023 R2, or FEKO 2022. The resonant frequencies $f_{m}^{i}$ under the influence of mutual coupling and ${∆}_{RCS}^{i}$ must be ascertained for use in Step 6.
- Step 6: Evaluate the level of deviation of the values ${∆}_{f}^{i}$ and ${∆}_{RCS}^{i}$ and compare with the threshold in Step 3. If the level of deviation satisfies the specified requirements, the design parameters of the tag are obtained, and the design process is completed (Step 8). Otherwise, this deviation is taken as the input for the optimization algorithm to propose another set of design parameters.
- Step 7: Deploy the optimization algorithm for the design parameters. This algorithm is based on the deviation to propose new sets of design parameters, which are then re-evaluated according to Step 5 until a satisfactory result is achieved.

According to the proposed design process, Steps 5–7 determine whether the tag design satisfies the coding requirements. The simulation of a tag structure is usually performed in minutes; hence, this optimization design process needs to converge after a sufficiently small number of simulations to be time efficient. In addition, for a small $\varepsilon_{f}^{i}$ and large ${∆}_{RCS}^{i}$, in order to converge, an adequate and highly effective optimization algorithm is required.

For the class of optimization problems where the relationship between the output and inputs cannot be modelled as a mathematical function, a heuristic approach is advantageous, in which the most well-known algorithms are the genetic algorithm (GA) [28], ant colony optimization (ACO) algorithm [29], and particle swarm optimization (PSO) algorithm [30]. With regard to complexity, convergence time, stability, and ability to globally optimize [31], PSO is used in this study. Applying PSO to Steps 5–7, the number of particles in the population corresponds to the number of proposed combinations of design parameters based on the theoretically calculated values in Step 4. The search space is defined by the bounds of the design parameters. The process of searching in different locations involves trials with different combinations. The objective function indicates the degree of satisfaction with the encoding requirements as a function of $\varepsilon_{f}^{i}$ and ${∆}_{RCS}^{i}$. To implement PSO, the jth particle in the swarm is characterized by two parameters: position $x_{j}^{k}$ and velocity $v_{j}^{k}$, where k is the iteration number. Position $x_{j}^{k}$ corresponds to the jth parameter combination, and $v_{j}^{k}$ indicates how to update $x_{j}^{k}$ in the next iteration, as follows:

(5a) $$v_{j}^{k+1}=\omega v_{j}^{k}+c_{1}r_{1}\left(P_{j}^{k}-x_{j}^{k}\right)+c_{2}r_{2}\left(G^{k}-x_{j}^{k}\right)$$

(5b) $$x_{j}^{k+1}=x_{j}^{k}+v_{j}^{k+1}$$

where $P_{j}^{k}$ is the best local position of the j^th^ particle up to iteration k, $G^{k}$ is the best global position of the entire swarm up to iteration k, ω is the constant inertia coefficient, $c_{1}$ and $c_{2}$ are constant acceleration coefficients ranging from 1.5 to 2.5, and $r_{1}$ and $r_{2}$ are varying random numbers in the range $\left[0,\;1\right]$.

In this study, MATLAB 2024a is used to implement PSO and continuously invokes CST to update the tag design parameters, conduct simulations, and collect the output. The total number of tried combinations in the entire process is important because the time taken to simulate a structure is usually in minutes. As shown in Figure 4, the initial frequency deviations of the elements are not the same, because the impact of mutual coupling differs for each element. Accordingly, for elements that are strongly affected by mutual coupling, it is necessary to adjust the design parameters more than for other elements, and vice versa. To reflect this statement, objective function Φ is designed to include the initial frequency error ${∆}_{f}^{i}$ in the weight. Moreover, the objective function should be large for frequency deviations located outside the range defined by $\varepsilon_{f}^{i}$, and similarly, when the value of ${∆}_{RCS}^{i}$ does not reach the threshold to differentiate extreme points. This helps the PSO to re-orient itself to other search areas. From this discussion, the objective function in this study is proposed as follows:

(6) $$\Phi =\sum_{i=1}^{N}\left|\frac{{∆}_{f}^{i}}{min\left\{{∆}_{f}^{i}\right\}}\right|\times {\left(f_{m}^{i}-f_{i}\right)}^{4}-\sum_{i=1}^{N-1}{\left({∆}_{RCS}^{i}\right)}^{2}$$

where N is the number of resonant frequencies of the tag.

Another important factor that directly affects the optimization time of the proposed design method is the initial particles. In the standard PSO [20,30] method, the initial particles are generated randomly within the search space, and the number of particles and iterations must be sufficiently large for the algorithm to converge to the global optimum value. Meanwhile, the number of particles may reach hundreds, and the number of iterations can increase to thousands depending on the complexity of the problem. It is irrelevant to apply this principle to the tag design problem because the evaluation of the objective function is time consuming, owing to the simulation. To address this problem, we propose finding high-quality particles and applying them as the initial ones. This helps the PSO converge more quickly.

To achieve this, a possible solution is using the set of theoretical design parameters calculated in Step 4, and the distance ratio between them to determine the initial particles. This is reasonable, because the theoretical design parameters should be close to the optimum value. Furthermore, the frequency deviation is caused by the mutual coupling between the resonant elements. However, this method is only applicable to cases where the design conditions are not too challenging, specifically when there are few resonant elements and the spacing between frequencies is large. Under these conditions, the impact of mutual coupling does not significantly shift the frequency from the threshold $\varepsilon_{f}^{i}$. In other words, with some initial particles close to the optimum value, the algorithm can find a satisfactory parameter set; however, this may only be a local optimum value. To overcome this problem, it is essential to specify more initial particles that are close to different local optima. The proposed solution is to utilize the Taguchi method (TM) [21] to find them.

The TM was developed based on the concept of orthogonal arrays ${OA}_{c}\left(s^{r}\right)$ [32], where c is the number of columns and number of experiments needed to find the optimal value, r is the number of rows and number of design parameters of the tag, s is the number of value levels of the parameters, and t is a parameter related to the repeatability in the columns that the orthogonal array must satisfy. It should be noted that in the experimental design methodology, the parameter variables are not continuous but have discrete levels. The important significance of this method is that c is much smaller than the number of conventional experiments required to find the optimal value, which is $s^{r}$. For instance, if an orthogonal array ${OA}_{27}\left(3^{13}\right)$ is applied to 13 variables, each with three levels, then the number of experiments is 27, rather than $13^{3}=2197$. Although the results obtained using this method are not optimal, they are generally good. To obtain multiple different candidate initial particles for PSO using the TM, it is vital to bring up many different levels for each design parameter. In the following section, the application of the proposed method to the design of a number of tag structures with selected frequency combinations is presented.

## 3. Sample Design Case Studies

Chipless RFID tags are often designed for the UWB frequency range from 3.1 to 10.6 (GHz) due to their short wavelength, thereby reducing the size of the tag. In addition, the UWB frequency range is currently being developed for indoor positioning applications, so many designs for this frequency range are used. In this study, we choose the frequency range [6.0, 8.0] (GHz) as the middle range of the UWB frequency range with the purpose of being able to expand the coding frequencies to both sides of the entire UWB frequency range in the future. The smaller the encoding frequency difference selected, the more data can be encoded within the initially selected frequency interval. However, the smaller the frequency difference, the easier it is for inductance to distort the tag’s encoded frequency data. Besides, this difference also depends on the bandwidth at the resonant frequency of the encoding element, which means it depends on the quality coefficient of the material used to make the tag.

### 3.1. Tag Design for Frequency Combination [6.7, 6.9, 7.1, 7.3, 7.5] (GHz)

According to the design method proposed in Section 2, Step 1 is to choose the type of tag and resonant elements. As for backscattering chipless tags, among the element types that have been proposed in the literature, the two most commonly used are dipole load lines and slot resonators. These two types of highly conductive resonant elements are usually designed with letter shapes, such as I [27,33], C [34,35], L [36,37], M [38], U [23], and O [39,40] letters. They are integrated on the surface of a substrate material that is suitable for high-frequency environments. In terms of the signal power scattered back to the reader, the slot resonator receives greater power because the metal plane receives incoming electromagnetic waves with a wider area. This makes it easier to measure the RCS value of the tag. Therefore, the slot type is used to design the resonant elements in this study. The I-shaped slot is chosen because it directly suffers from mutual coupling in addition to its simplicity and ease of design. The design requirement is to compensate for the influence of mutual coupling to achieve selected resonant frequencies.

Step 2 selects the resonant frequency set used to encode the data in the range of the transceiver antenna, while the difference between the encoding resonant frequencies must be sufficiently small to guarantee the encoding capacity. In this study, 15 frequencies in the range [6.2 ÷ 7.6] (GHz) are designated and evenly distributed with a distance of $∆f_{s}^{i}=0.1\;$(GHz). In addition to selecting a set of frequencies, it is necessary to specify the encoding method. Following the discussion in Section 2, the FSC method is used so that an element can work at many frequencies, and five elements are used to encode these frequencies. However, each element can work at any frequency in the set to maximize the number of possible codes compared with the traditional FSC method. This coding enhancement is possible because of the proposed design method.

In Step 3, the allowed frequency deviation limit is calculated as $\varepsilon_{f}^{i}=∆f_{s}^{i}/2=0.05$ (GHz) and the RCS threshold value ${∆}_{RCS}^{i}$ is chosen to be 3 dBsm.

In Step 4, for I-shaped slot elements, there are two approaches for approximating the element length according to the desired frequency. The first approach considers the resonant slot as an independent half-wavelength dipole antenna [33]. The equation relating the frequency f with the slot length L, and the relative dielectric constant of the substrate material $\varepsilon_{r}$, is as follows:

(7) $$f=\frac{c\sqrt{\frac{2}{\varepsilon_{r}+1}}}{2L}$$

where c is the speed of light.

The second approach considers the resonant element as a resonant slot etched on a patch antenna covering the working frequency range with the same material as the tag [41]. The equations pertaining to the resonant frequency to the design parameters of the UWB patch antenna are as follows:

(8a) $$L=\frac{c\times B}{2\times f}\times \left[\frac{A}{B}-\ln\left(\frac{f\times h}{c}\right)\right]$$

(8b) $$A=1.045-0.365\times \ln\varepsilon_{r}+\frac{6.3\times d/h\times \varepsilon_{r}^{0.945}}{238.64+100\times d/h}$$

(8c) $$B=0.148-\frac{8.81\times \left(\varepsilon_{r}+0.95\right)}{100\times \varepsilon_{r}}$$

where λ is the wavelength of the resonant frequency, d is the slot width, and h is the substrate thickness, under the following conditions:

(9) $$2.2\leq \varepsilon_{r}\leq 3.8,\;0.006\leq h/\lambda \leq 0.06,\;0.0015\leq d/\lambda \leq 0.075$$

For an element on a tag without a fixed waveguide port as mentioned in Section 1, the above theoretical calculations provide only approximate results. In this study, the second approach is used for a backscattering chipless RFID system. To comply with the conditions in Equation (9), the substrate material for the tag is adopted as polyimide, with a dielectric constant $\varepsilon_{r}=3.5$, loss coefficient tanδ=0.0027, slot width d=0.5 (mm), substrate thickness h=0.1 (mm), and copper layer thickness of 0.089 (mm). This material has the advantages of a low price, flexibility, and temperature resistance. In addition, as this is not a specialized material for receiving and transmitting electromagnetic waves, it will become a beneficial feature if this designed tag can satisfy frequency encoding.

In the operating frequency range of 6 GHz to 8 GHz, with a wavelength λ=c/f<50 mm, the size of the tag structure can be selected as 30×30 (mm^2^) to make each dimension greater than the half wavelength.

**Figure 7:**
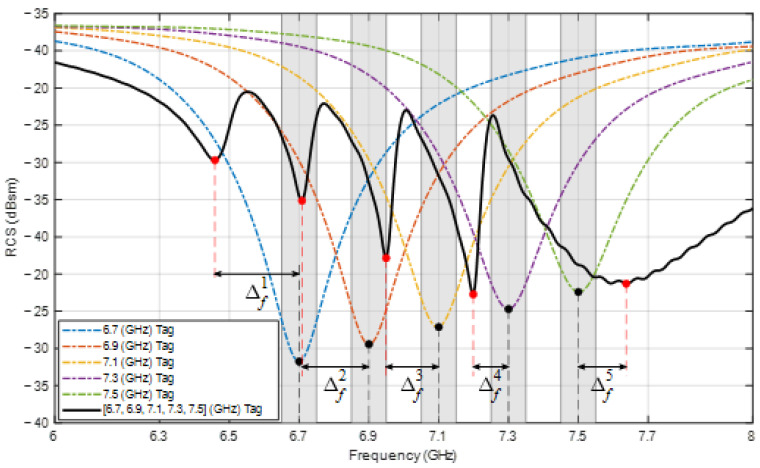
Simulated RCS response of the single-element tags and the tag combining all the elements.

The resonant frequencies chosen to design the tag using the proposed method are 6.7, 6.9, 7.1, 7.3, and 7.5 GHz, which are five frequencies evenly distributed in the set of fifteen selected encoding frequencies. Frequencies with the same difference of 0.2 GHz are chosen to set the simulation time and design in accordance with the configuration of the computer (Core i7 Gen13 3.6 GHz CPU, 32 GB RAM) that is used to run CST and at the same time they are sufficiently hard to demonstrate the generality of the proposed method. From these frequencies, the formulas in Equation (8) are used to approximate the element lengths. To obtain the correct resonant frequency response at the desired frequencies, it is necessary to conduct an investigation and adjustment, which in this case is not complicated, because only one element is present each time, there is no mutual coupling, and it can be achieved with any standard antenna design software. In this study, using the optimization feature in CST Studio Suite, Table 2 lists the obtained lengths of the elements corresponding to the selected frequencies. The RCS response of the designed single-element tags is shown in Figure 7 by dashed lines. It is apparent that the minima of the RCS match the corresponding encoding frequencies of the resonant elements.

**Figure 8:**
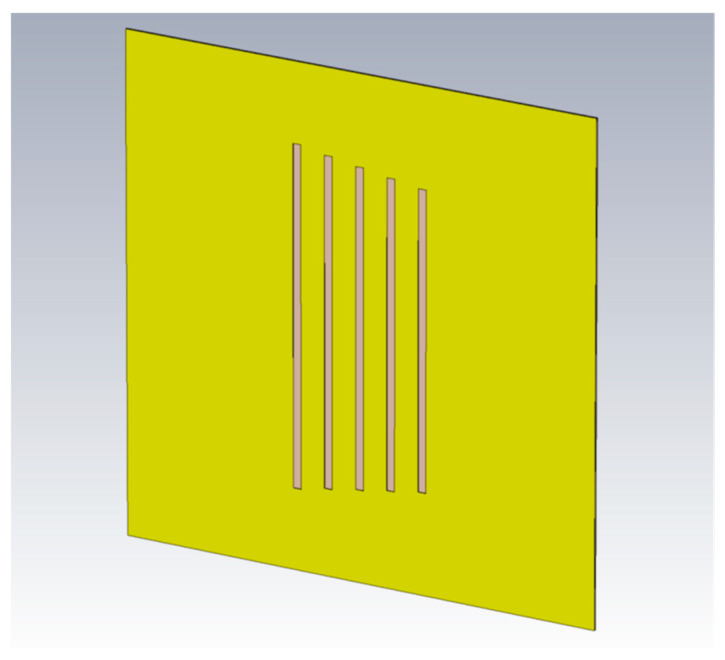
Tag combining all the 5 resonant elements without modification.

Steps 5–7 are performed according to the design process based on two PSO variations, with and without applying the Taguchi method for particle initialization. Therefore, it is necessary to define the coefficients of the proposed objective function in Equation (6). The tag structure shown in Figure 8, combining all the five resonant elements with the length in Table 2 without modification needs, is investigated, in which the spacing $S^{i,i+1}$ between the ith and $\left(i+1\right)$^th^ elements is chosen to be 2 mm, which is large enough to reduce the influence of mutual coupling from other resonant elements and small enough to integrate multiple elements to further enhance the encoding ability of the tag. As suggested in [18], the mutual coupling between two resonant elements only decreases when the relative distance between two elements exceeds five times the wavelength. The tag size of 30 mm in each direction is smaller than the wavelength of 43 mm of the center frequency of 7 GHz. If we consider the resonant elements as antennas, these antennas are in each other’s near field, so the mutual coupling effect is high and unavoidable. In this study, we choose a distance of 2 mm, with the main purpose being that the tag can integrate up to 15 resonant elements; further, it is a very challenging problem to showcase the effectiveness of the proposed method. It is also needed to provide a reminder that the proposed method does not aim to cancel out the mutual coupling effect, but to compensate it by adjusting the element design parameters.

The RCS response of this combined tag is shown in Figure 7 as a solid line. It can be observed that ${∆}_{RCS}^{i}$ satisfies the threshold of 3 dBsm for every frequency. However, none of the new frequencies $f_{m}^{i}$ is located within the limit $\varepsilon_{f}^{i}$ of 0.05 GHz from the original $f^{i}\;$frequencies. More specifically, the new frequencies are now 6.46, 6.71, 6.95, 7.20, and 7.64 GHz, which correspond to the highest frequency deviation of 0.24 GHz and the lowest frequency deviation of 0.10 GHz. Even worse, $f_{m}^{2}$ and $f_{m}^{3}$ are located in the accepted ranges of $f_{m}^{1}$ and $f_{m}^{2}$, respectively, which leads to defective data bits. Consequently, this tag structure does not satisfy the frequency encoding requirements, owing to the influence of mutual coupling. From this figure, it is possible to determine the initial frequency deviations ${∆}_{f}^{i}$ to calculate the weights of mutual coupling at the frequencies in the objective function in Equation (6).

From the input parameters in Table 2, the design parameter set subject to optimization includes five length deviations $∆L^{i}$ and four spacing deviations $∆S^{i,i+1}$. Regarding the bounds of these parameters, by considering the tag size limitation as well as manufacturing capabilities, the range ${\;\varepsilon }_{L}$ for length deviation is chosen to be $\pm \frac{L^{1}-L^{5}}{2}=\pm 1.24$ (mm), and the range $\varepsilon_{S}$ for element spacing is chosen to be [−1.25 ÷ 2.0] (mm).

Regarding the PSO parameters, the number of particles and the maximum iterations are chosen to be 60 and 300, respectively, and coefficients $c_{1}$ and $c_{2}$ are both set as 2.1. The acceleration coefficient ω directly affects the difference in the value of each particle after each iteration, thereby directly influencing the convergence rate. To converge quickly and accurately, the coefficient ω needs to gradually decrease after each iteration; hence, the update value is selected in the range [0.4 ÷ 0.9]. In this case, the calculated parameters are used to initialize the PSO particles. In other words, $∆L^{i}$ and $∆S^{i,i+1}$ are initially set to 0. All tags are automatically designed and simulated using CST software 2024 to determine the corresponding objective values. This PSO algorithm merely stops when it finds a set of parameters that meet the requirements of $\varepsilon_{f}^{i}$ and ${∆}_{RCS}^{i}$, or when it reaches the maximum number of iterations of 300.

When the TM is applied with nine parameters, the orthogonal array ${OA}_{27}\left(3^{9}\right)$ is used. In other words, with three levels per parameter, 27 simulations are necessary to find the particle used in the initialization. To initialize the entire set of particles, it is necessary to perform the TM with many initial parameter limit cases. For this design problem, 10 cases of changing parameter limiters ($\varepsilon_{L}$, $\varepsilon_{S}$) are proposed, and the results of the 10 sets of parameters are summarized in Table 3. These sets are combined with the theoretical parameter set and random parameter sets to initialize the 60 initial particles used in the PSO method.

**Figure 9:**
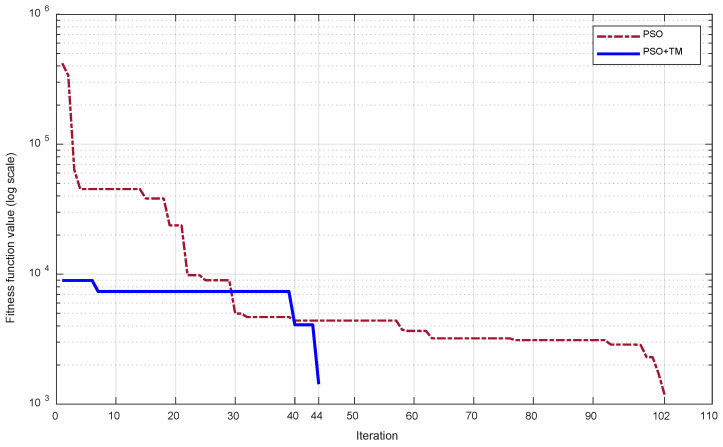
Convergence process of the objective function for frequencies [6.7, 6.9, 7.1, 7.3, 7.5] (GHz).

With the above setup, the optimization progress using PSO characterized by the best objective value Φ obtained in each iteration is shown in Figure 9 for the two cases, with and without the TM. It can be observed that when only PSO is used, the requirements are met after 102 iterations and Φ attains 1185. When PSO and the TM are used together, PSO starts with better initial particles whose objective values are smaller, and the requirements are met after 44 iterations and Φ attains 1425. With the help of the TM, PSO can find the optimized parameters in a much shorter time.

**Figure 10:**
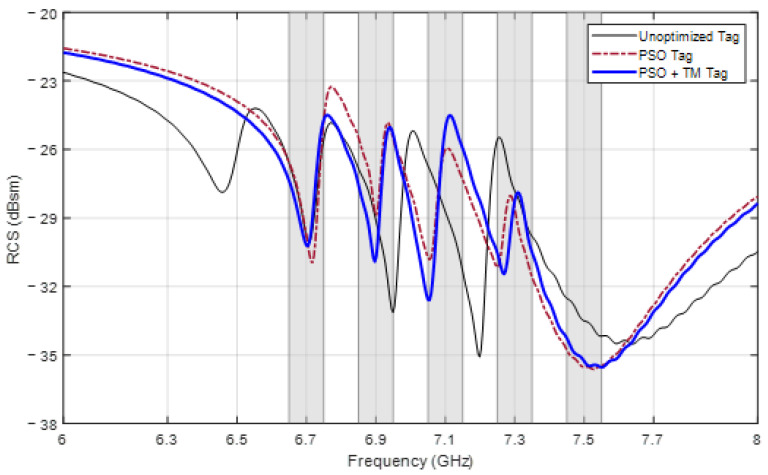
RCS response of tags designed for frequencies [6.7, 6.9, 7.1, 7.3, 7.5] (GHz).

The resultant design parameters and resonant frequencies for both cases are presented in Table 4, and the RCS responses of the optimized tags are shown in Figure 10. The tag optimized with PSO alone resonates at frequencies of 6.72, 6.90, 7.05, 7.25, and 7.53 GHz, for which the mean frequency error is 0.03 GHz. For the tag optimized with PSO and the TM, the resonant frequencies include 6.70, 6.90, 7.05, 7.27, and 7.55 GHz with a mean error of 0.026 GHz. From the figure, it can be observed that all the requirements for ${∆}_{f}^{i}$ and ${∆}_{RCS}^{i}$ are satisfied in both cases.

### 3.2. Tag Design for Frequency Combination [6.6, 6.8, 7.0, 7.2, 7.4] (GHz)

**Figure 11:**
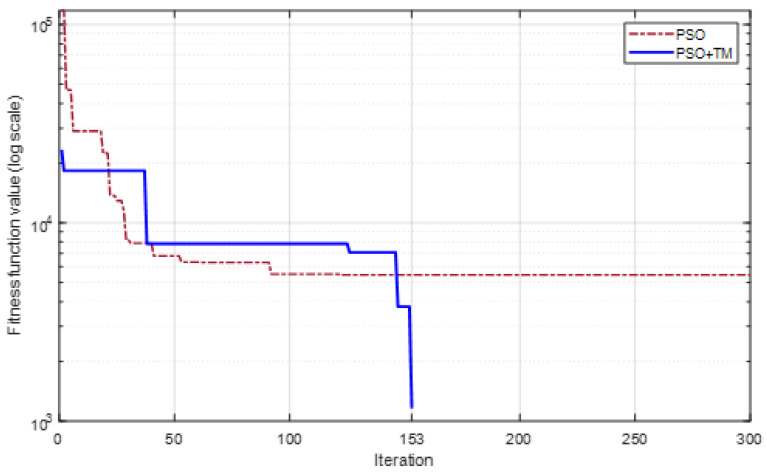
Convergence process of the objective function for frequencies [6.6, 6.8, 7.0, 7.2, 7.4] (GHz).

To further evaluate the proposed design methodology and compare the two methods of initializing particles, another combination of encoding frequencies is selected to design the tag as 6.6, 6.8, 7.0, 7.2, and 7.4 GHz. The convergence process for both initialization methods for this combination is illustrated in Figure 11. According to this figure, when using a theoretically designed combination and then adjusting it as an initial particle, the PSO algorithm does not achieve satisfactory results after running 300 iterations for the design requirements. In addition, it can be seen from the iterations from 100 to 300 that no better value is achieved. This can be interpreted as follows: among the initial particles, only a few elements are close to a local optimum value, and PSO is not capable of walking over it to find the global optimum within the limit number of iterations. When additional TM-initialized particles are used, the optimized parameter set is achieved after 153 iterations.

**Figure 12:**
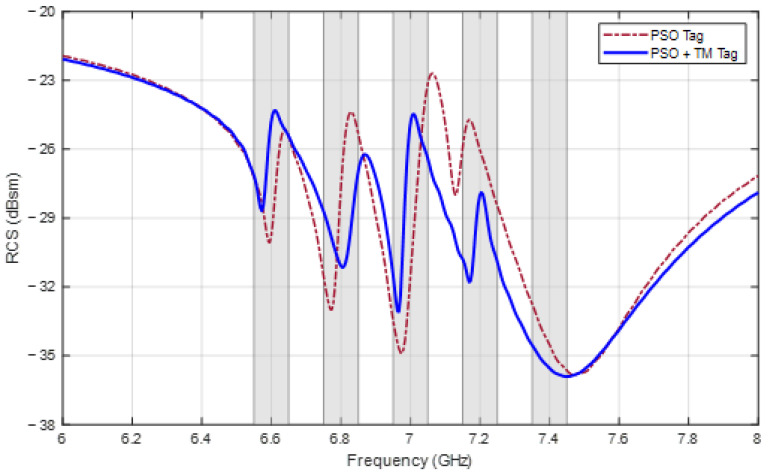
RCS response of tags designed for frequencies [6.6, 6.8, 7.0, 7.2, 7.4] (GHz).

Table 5 shows the frequencies and design parameters achieved by the two methods, and the RCS responses of the two tags are shown in Figure 12. From the figure, all the ${∆}_{RCS}^{i}$ values are also larger than the limit of 3 dBsm in both cases. However, with respect to the frequency, the best combination that PSO finds is 6.59, 6.77, 6.98, 7.13, and 7.47 GHz with a mean absolute error of 0.04 GHz and has the last two frequencies that are not located in the accepted ranges. In the other case, when the TM is applied, the obtained frequencies are 6.57, 6.81, 6.97, 7.17, and 7.45 GHz with a mean error of 0.03 GHz, and all of them are satisfactory.

In conclusion, this example demonstrates that the proposed design methodology is also useful for overcoming the cases where PSO has trouble and is stuck in generating good-quality initial parameter combinations.

### 3.3. Tag Design for Frequency Combination [6.6, 6.8, 6.9, 7.2, 7.4] (GHz)

**Figure 13:**
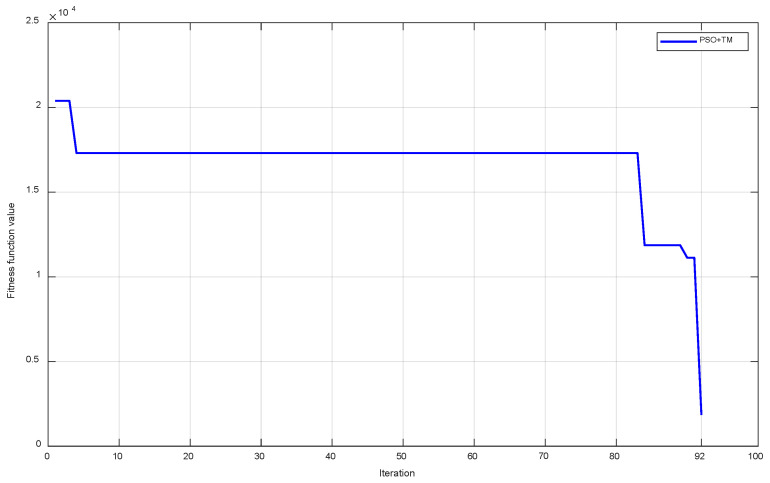
Convergence process of the objective function for frequencies [6.6, 6.8, 6.9, 7.2, 7.4] (GHz).

In this subsection, a combination of five frequencies, 6.6, 6.8, 6.9, 7.2, and 7.4 GHz, is used in the tag design. It can be noticed that this combination differs from the combination in Section 3.2 by the third frequency, which is now closer to the second one in order to make it more challenging. This smaller frequency discrimination between 6.8 GHz and 6.9 GHz obviously increases the influence of mutual coupling, which cause more difficulty in achieving optimized parameters for the tag design. However, the influence of mutual coupling between the two encoding frequencies, 6.9 GHz and 7.2 GHz, is reduced simultaneously. The TM is also applied to initialize PSO. The convergence process of the objective function is illustrated in Figure 13. The requirements are satisfied after 92 iterations when the objective function reaches 1840.

**Figure 14:**
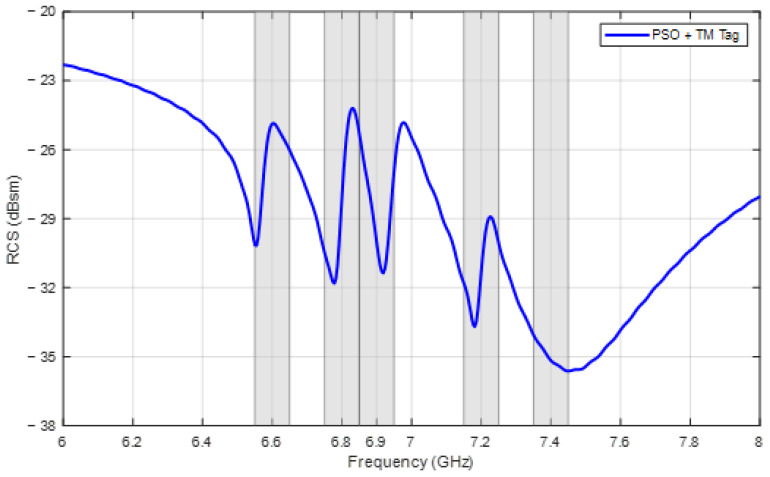
RCS response of tag designed for frequencies [6.6, 6.8, 6.9, 7.2, 7.4] (GHz).

The optimized parameters for this frequency set are listed in Table 6. The RCS response of the corresponding tag is displayed in Figure 14, revealing that the achieved resonant frequencies are 6.56, 6.78, 6.92, 7.18, and 7.44 GHz, which are all located in the accepted encoding frequency ranges with a threshold of 0.05 GHz.

## 4. Experimental Results

**Figure 15:**
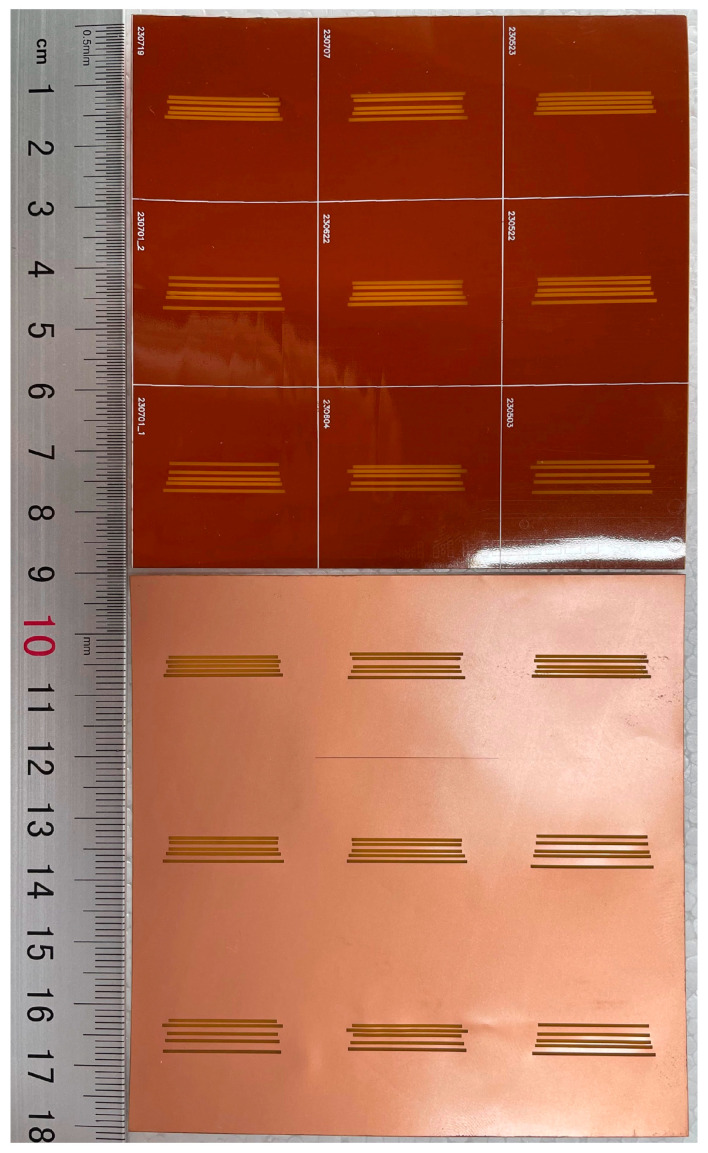
Fabricated tag prototypes.

**Figure 16:**
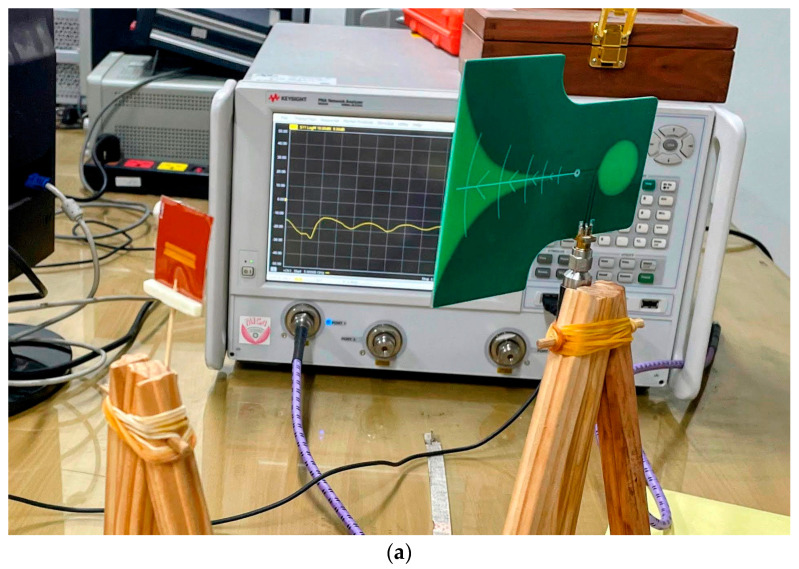

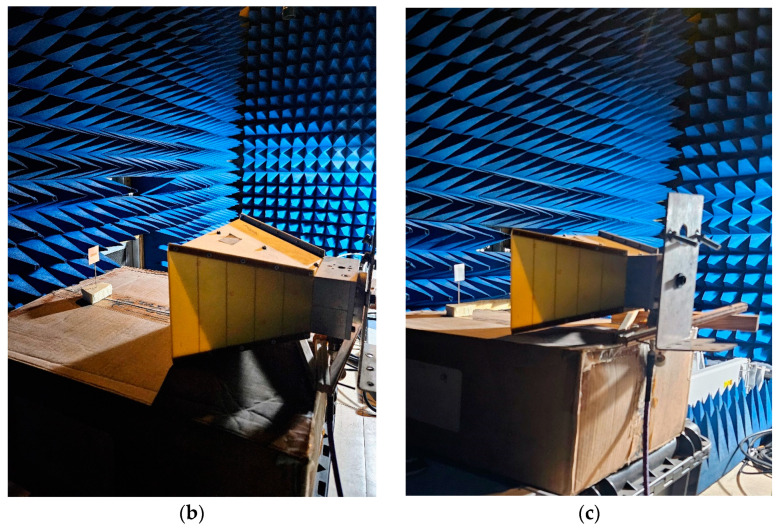
Tag RCS measurement system: (**a**) in ambient chamber with a designed tag; (**b**) in anechoic chamber with a designed tag; and (**c**) in anechoic chamber with a reference tag.

To verify the simulation results for optimally designed tags using the proposed method, sample tags are fabricated using the selected materials and parameters, as shown in Figure 15. Two RCS measurement systems are set up with a vector network analyzer (VNA) working in the frequency range from 6.0 to 8.0 GHz, one in an ambient measurement chamber, and another in an anechoic chamber, as shown in Figure 16. In both systems, a UWB antenna is used simultaneously to transmit electromagnetic signals to the tag and receive backscattered electromagnetic waves. A reference tag is also used in the measurements so that the RCS response can later be calibrated using Equation (4). One antenna is used to avoid mutual coupling between the pair of transceiver antennas in the two-antenna measurement mode. The RCS response of the tag according to the frequency is calculated using the measured reflection coefficient $S_{11}$ with the help of Equation (4). The distance from the antenna to the tag is 22 (cm), in the far field, according to the specifications of the antenna in use.

**Figure 17:**
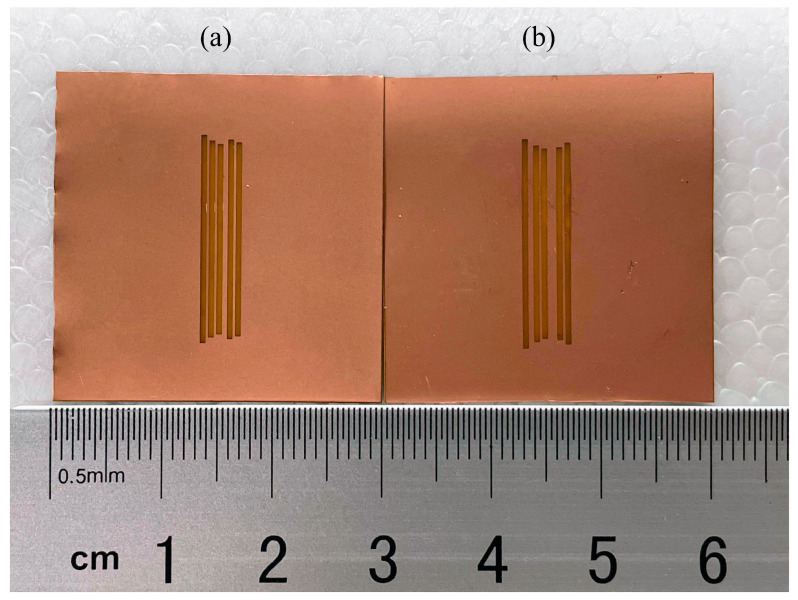
Tags fabricated with parameter sets optimized for frequencies [6.7, 6.9, 7.1, 7.3, 7.5] (GHz) using (**a**) PSO only, and using (**b**) PSO with TM.

**Figure 18:**
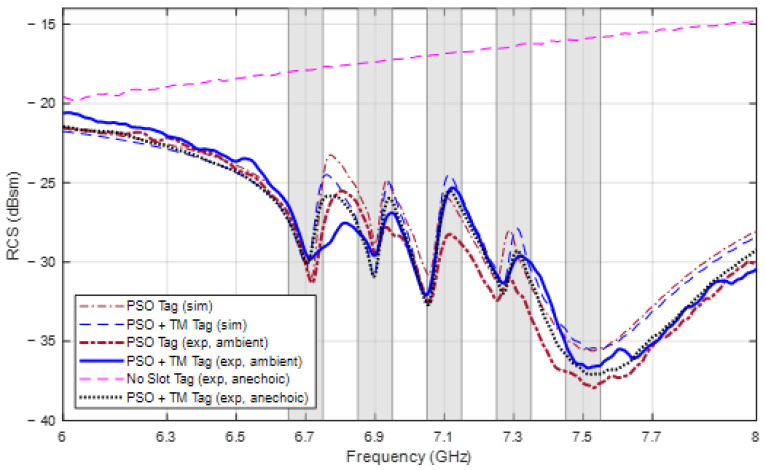
Simulated and measured RCS response of the two tags designed for frequencies 6.7, 6.9, 7.1, 7.3, and 7.5 GHz, and a reference tag.

Figure 17 shows the two tags fabricated using the two parameter sets from Table 4 optimized for frequencies 6.7, 6.9, 7.1, 7.3, and 7.5 GHz, in which the tag on the left is optimized using PSO without the TM, whereas the tag on the right corresponds to the optimization that includes the TM. The measured RCS responses of these two tags are shown in Figure 18 along with the simulated response and the response of a tag without elements in the anechoic chamber. The corresponding resonant frequencies, as shown in Table 7, are all located within the accepted range bounded by the threshold of 0.05 GHz from the selected frequencies, and they are all very close to the simulated values. Since the requirements are satisfied, the data bits can be properly decoded by the reader.

Even under ambient measurement conditions where frequencies are subject to electromagnetic interference from noise emitted by many devices and their signals reflected by objects in the surrounding environment, this noise can be considered as white noise which makes the measured RCS magnitude not accurate, but its form is still reserved and the frequencies are reliable. Another factor that affects the measured RCS is that the gain properties of the antenna used in the working frequency range are not as accurate as those in the simulation. For this reason, the measured RCS response in both chambers still has a similar shape to the simulated response, but at the second and fourth frequencies, ${∆}_{RCS}^{i}$, when measured in the ambient chamber, is smaller than the required difference of 3 dBsm. In this case, the smallest RCS difference is only 1.47 GHz at the fourth frequency of both tags, but the two extreme RCS values are still distinct.

**Figure 19:**
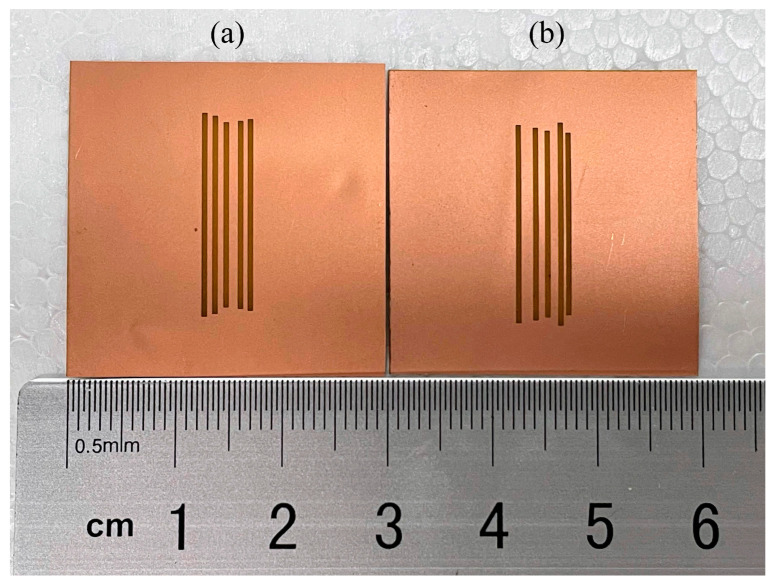
Tags fabricated with parameter sets optimized for frequencies: (**a**) [6.6, 6.8, 7.0, 7.2, 7.4] (GHz), and (**b**) [6.6, 6.8, 6.9, 7.2, 7.4] (GHz).

**Figure 20:**
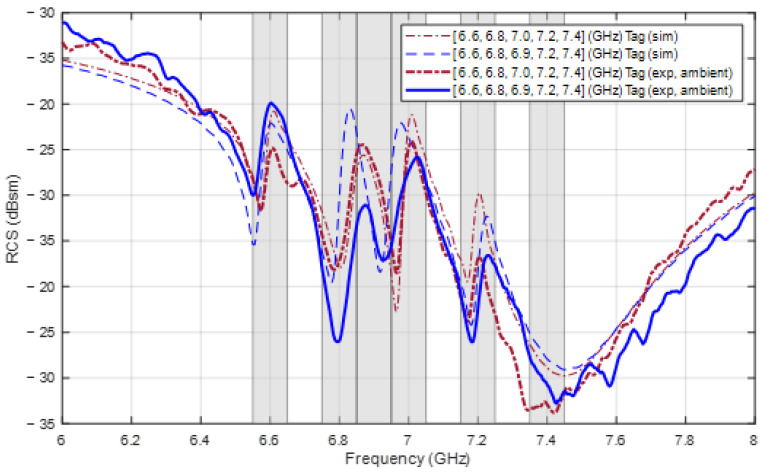
Simulated and measured RCS response of the two tags designed for frequencies [6.6, 6.8, 7.0, 7.2, 7.4] (GHz) and [6.6, 6.8, 6.9, 7.2, 7.4] (GHz).

Next, two other tags fabricated with the parameters in Table 5 and Table 6, optimized for frequencies 6.6, 6.8, 7.0, 7.2, and 7.4 GHz, and 6.6, 6.8, 6.9, 7.2, and 7.4 GHz, both using PSO initialized with the TM, are shown in Figure 19a,b, respectively. The RCS responses of these two tags measured in the anechoic chamber are shown in Figure 20 along with the simulated response for comparison, while the measured frequencies are listed in Table 8. It can be seen that the frequencies at which resonance occurs are all satisfactory, and they are almost the same as those provided by the simulation. However, the discrimination of the ${∆}_{RCS}^{i}$ value at some frequencies is not as high as that in the simulation results. This problem is caused by the measurements in an ambient chamber.

## 5. Conclusions

This study focuses on the issue of the low reliability of frequency-encoded data in chipless RFID technology. Two principal factors that reduce reliability are the deviation of the encoding frequency when modifying the encoding data of the tag and the amplitude at the extreme points of the RCS value. The direct cause of these factors is that the mutual coupling phenomenon between the resonant elements on different tags differ. Starting from the fact that current research has not yet developed a method to completely compensate for this effect, a new design approach is proposed in this study, using an appropriate optimization algorithm that is capable of providing tags with highly reliable data encryption. The novelty of the proposed approach is that it allows the free pre-selection of the encoding frequencies and then proceeds with the optimal design of the resonant elements, in contrast to the usual way of designing the corresponding resonant elements first and then adjusting them to obtain the frequencies that are often unpredictable. This not only makes tag design more proactive but also allows for an increase in the encoding capacity of the tags.

The design process of the proposed method is presented step by step, in which the analysis to apply the PSO and Taguchi optimization algorithms is also presented in detail. The tags were designed and fabricated by selecting specific frequency encoding combinations. The simulation and measurement results proved the correctness of the method, as well as the role of the initialization of the initial elements using the Taguchi method. Since the proposed approach does not take into account the specificity of the I-slotted tag structure used in the case studies, it is general and in principle can be applied to other types of tag structures. Another research perspective of this approach is the application in other tag and antenna design problems, for which polarization dependency reduction is an example.

## Figures and Tables

**Table 1:** Frequencies produced by resonant elements when working individually and when present all together on the same tag.

Element Number	Original Frequency (GHz)	Shifted Frequency (GHz)	Frequency Deviation (GHz)	Element Number	Original Frequency (GHz)	Shifted Frequency (GHz)	Frequency Deviation (GHz)
1	4.01	3.71	−0.30	11	4.67	4.54	−0.13
2	4.07	4.04	−0.03	12	4.71	4.60	−0.11
3	4.11	4.11	0.00	13	4.71	4.65	−0.06
4	4.19	4.17	−0.02	14	4.74	4.70	−0.04
5	4.26	4.22	−0.04	15	4.76	4.77	0.01
6	4.34	4.28	−0.06	16	4.76	4.82	0.06
7	4.42	4.33	−0.09	17	4.79	4.89	0.10
8	4.50	4.38	−0.12	18	4.80	4.94	0.14
9	4.55	4.44	−0.11	19	4.86	5.02	0.16
10	4.61	4.48	−0.13	20	4.92	6.00	1.08

**Table 2:** Parameters of single-element tags after tuning.

Resonant frequency (GHz)	$f^{1}$	$f^{2}$	$f^{3}$	$f^{4}$	$f^{5}$
	6.7	6.9	7.1	7.3	7.5
Element length (mm)	$L^{1}$	$L^{2}$	$L^{3}$	$L^{4}$	$L^{5}$
	19.96	19.31	18.71	18.12	17.57

**Table 3:** Initial parameter sets proposed with help of the TM (mm).

${∆L}^{1}$	$∆L^{2}$	$∆L^{3}$	$∆L^{4}$	$∆L^{5}$	${∆S}^{1,2}$	${∆S}^{2,3}$	$∆S^{3,4}$	$∆S^{4,5}$
−0.28	−0.42	−0.31	−0.34	−0.45	0.48	−0.81	−1.15	−1.20
−0.25	−0.28	−0.28	−0.24	−0.18	−0.34	−1.06	−1.05	1.11
0.16	−0.01	−0.11	−0.72	−0.68	1.07	−0.18	−0.61	−0.46
−0.14	−0.35	−0.58	−0.35	−0.04	0.20	−0.84	−1.06	−1.17
−0.35	−1.10	−0.03	−0.42	−0.35	0.07	−0.96	−1.07	−0.95
−0.28	−0.49	−0.41	−0.25	−0.03	1.03	−1.25	−1.23	−1.04
−0.21	−0.24	−0.18	−0.30	−0.51	−0.11	−0.94	0.19	−0.94
−0.25	−0.75	−0.88	−0.63	−0.86	1.49	−1.25	−1.25	−1.23
−0.24	−0.12	−0.94	0.13	−0.26	−0.10	−0.75	−0.68	−1.13
−0.52	−0.39	−0.44	−0.26	0.00	−0.45	−0.63	−0.63	−0.56

**Table 4:** Optimization results for frequencies [6.7, 6.9, 7.1, 7.3, 7.5] (GHz).

Desired resonant frequency (GHz)	$f^{1}$	$f^{2}$	$f^{3}$	$f^{4}$	$f^{5}$
	6.7	6.9	7.1	7.3	7.5
Achieved frequency (GHz)	$f_{m}^{1}$	$f_{m}^{2}$	$f_{m}^{3}$	$f_{m}^{4}$	$f_{m}^{5}$
PSO alone	6.72	6.90	7.05	7.25	7.53
PSO and TM	6.70	6.90	7.05	7.27	7.55
Element length adjustment (mm)	${∆L}^{1}$	$∆L^{2}$	$∆L^{3}$	$∆L^{4}$	$∆L^{5}$
PSO alone	−0.82	−1.20	−1.20	0.24	0.28
PSO and TM	−0.68	−1.195	−1.195	−0.38	0.99
Element length (mm)	$L^{1}$	$L^{2}$	$L^{3}$	$L^{4}$	$L^{5}$
PSO alone	19.14	18.11	17.52	18.36	17.85
PSO and TM	19.28	18.11	17.52	17.74	18.56
Element distance adjustment (mm)	${∆S}^{1,2}$	${∆S}^{2,3}$	$∆S^{3,4}$	$∆S^{4,5}$	
PSO alone	−1.25	−1.25	−1.02	−1.25	
PSO and TM	−0.98	−1.25	−0.62	−1.25	
Element distance (mm)	$S^{1,2}$	$S^{2,3}$	$∆S^{3,4}$	$S^{4,5}$	
PSO alone	0.75	0.75	0.98	0.75	
PSO and TM	1.02	0.75	1.38	0.75	

**Table 5:** Optimization results for frequencies [6.6, 6.8, 7.0, 7.2, 7.4] (GHz).

Desired resonant frequency (GHz)	$f^{1}$	$f^{2}$	$f^{3}$	$f^{4}$	$f^{5}$
	6.6	6.8	7.0	7.2	7.4
Achieved frequency (GHz)	$f_{m}^{1}$	$f_{m}^{2}$	$f_{m}^{3}$	$f_{m}^{4}$	$f_{m}^{5}$
PSO alone	6.59	6.77	6.98	7.13	7.47
PSO and TM	6.57	6.81	6.97	7.17	7.45
Element length adjustment (mm)	${∆L}^{1}$	$∆L^{2}$	$∆L^{3}$	$∆L^{4}$	$∆L^{5}$
PSO alone	−0.84	−1.24	−1.18	−0.75	1.24
PSO and TM	−0.78	−0.62	−1.24	−0.35	0.57
Element length (mm)	$L^{1}$	$L^{2}$	$L^{3}$	$L^{4}$	$L^{5}$
PSO alone	19.48	18.39	17.84	17.66	19.08
PSO and TM	19.54	19.01	17.78	18.06	18.41
Element distance adjustment (mm)	${∆S}^{1,2}$	${∆S}^{2,3}$	$∆S^{3,4}$	$∆S^{4,5}$	
PSO alone	−1.25	−1.25	−1.25	−1.25	
PSO and TM	−1.00	−0.95	−0.64	−1.07	
Element distance (mm)	$S^{1,2}$	$S^{2,3}$	$∆S^{3,4}$	$S^{4,5}$	
PSO alone	0.75	0.75	0.75	0.75	
PSO and TM	1	1.05	1.36	0.93	

**Table 6:** Optimization results for frequencies [6.6, 6.8, 6.9, 7.2, 7.4] (GHz).

Resonant frequency (GHz)	$f^{1}$	$f^{2}$	$f^{3}$	$f^{4}$	$f^{5}$
	6.6	6.8	6.9	7.2	7.4
Element length adjustment (mm)	${∆L}^{1}$	$∆L^{2}$	$∆L^{3}$	$∆L^{4}$	$∆L^{5}$
	−1.15	−1	−1.24	1.24	−0.18
Element length (mm)	$L^{1}$	$L^{2}$	$L^{3}$	$L^{4}$	$L^{5}$
	19.17	18.63	18.07	19.65	17.66
Element spacing adjustment (mm)	${∆S}^{1,2}$	${∆S}^{2,3}$	$∆S^{3,4}$	$∆S^{4,5}$	
	−0.35	−0.86	−0.73	−1.25	
Element spacing (mm)	$S^{1,2}$	$S^{2,3}$	$S^{3,4}$	$S^{4,5}$	
	1.65	1.14	1.27	0.75	

**Table 7:** Measured frequencies of the two tags designed for frequencies [6.7, 6.9, 7.1, 7.3, 7.5] (GHz).

Desired resonant frequency (GHz)	$f^{1}$	$f^{2}$	$f^{3}$	$f^{4}$	$f^{5}$
	6.7	6.9	7.1	7.3	7.5
Measured frequency with PSO alone (GHz) in ambient chamber	$f_{m}^{1}$	$f_{m}^{2}$	$f_{m}^{3}$	$f_{m}^{4}$	$f_{m}^{5}$
	6.72	6.90	7.06	7.25	7.53
Measured frequency with PSO and TM (GHz) in ambient chamber	$f_{m}^{1}$	$f_{m}^{2}$	$f_{m}^{3}$	$f_{m}^{4}$	$f_{m}^{5}$
	6.71	6.90	7.05	7.27	7.51
Measured frequency with PSO and TM (GHz) in anechoic chamber	$f_{m}^{1}$	$f_{m}^{2}$	$f_{m}^{3}$	$f_{m}^{4}$	$f_{m}^{5}$
	6.70	6.90	7.05	7.27	7.52

**Table 8:** Measured frequencies of the two tags designed for frequencies [6.6, 6.8, 7.0, 7.2, 7.4] (GHz) and [6.6, 6.8, 6.9, 7.2, 7.4] (GHz).

[6.6, 6.8, 7.0, 7.2, 7.4] (GHz) tag	Desired frequency (GHz)	$f^{1}$	$f^{2}$	$f^{3}$	$f^{4}$	$f^{5}$
		6.6	6.8	7.0	7.2	7.4
	Measured frequency (GHz)	$f_{m}^{1}$	$f_{m}^{2}$	$f_{m}^{3}$	$f_{m}^{4}$	$f_{m}^{5}$
		6.57	6.79	6.96	7.17	7.42
[6.6, 6.8, 6.9, 7.2, 7.4] (GHz) tag	Desired frequency (GHz)	$f^{1}$	$f^{2}$	$f^{3}$	$f^{4}$	$f^{5}$
		6.6	6.8	6.9	7.2	7.4
	Measured frequency (GHz)	$f_{m}^{1}$	$f_{m}^{2}$	$f_{m}^{3}$	$f_{m}^{4}$	$f_{m}^{5}$
		6.56	6.80	6.92	7.19	7.43

## Data Availability

Data are contained within the article.
